# Learning three-dimensional aortic root assessment based on sparse annotations

**DOI:** 10.1117/1.JMI.11.4.044504

**Published:** 2024-07-30

**Authors:** Johanna Brosig, Nina Krüger, Inna Khasyanova, Isaac Wamala, Matthias Ivantsits, Simon Sündermann, Jörg Kempfert, Stefan Heldmann, Anja Hennemuth

**Affiliations:** a Fraunhofer Institute for Digital Medicine MEVIS, Bremen, Germany; b Institute of Computer-Assisted Cardiovascular Medicine, Deutsches Herzzentrum der Charité, Berlin, Germany; c Charité-Universitätsmedizin Berlin, Berlin, Germany; d Deutsches Herzzentrum der Charité, Department of Cardiothoracic and Vascular Surgery, Berlin, Germany; e DZHK (German Center for Cardiovascular Research), Partner Site Berlin, Berlin, Germany; f University Medical Center Hamburg-Eppendorf, Department of Diagnostic and Interventional Radiology and Nuclear Medicine, Hamburg, Germany

**Keywords:** left ventricular outflow tract, segmentation, annotation, transcatheter aortic valve implantation, aortic root

## Abstract

**Purpose:**

Analyzing the anatomy of the aorta and left ventricular outflow tract (LVOT) is crucial for risk assessment and planning of transcatheter aortic valve implantation (TAVI). A comprehensive analysis of the aortic root and LVOT requires the extraction of the patient-individual anatomy via segmentation. Deep learning has shown good performance on various segmentation tasks. If this is formulated as a supervised problem, large amounts of annotated data are required for training. Therefore, minimizing the annotation complexity is desirable.

**Approach:**

We propose two-dimensional (2D) cross-sectional annotation and point cloud-based surface reconstruction to train a fully automatic 3D segmentation network for the aortic root and the LVOT. Our sparse annotation scheme enables easy and fast training data generation for tubular structures such as the aortic root. From the segmentation results, we derive clinically relevant parameters for TAVI planning.

**Results:**

The proposed 2D cross-sectional annotation results in high inter-observer agreement [Dice similarity coefficient (DSC): 0.94]. The segmentation model achieves a DSC of 0.90 and an average surface distance of 0.96 mm. Our approach achieves an aortic annulus maximum diameter difference between prediction and annotation of 0.45 mm (inter-observer variance: 0.25 mm).

**Conclusions:**

The presented approach facilitates reproducible annotations. The annotations allow for training accurate segmentation models of the aortic root and LVOT. The segmentation results facilitate reproducible and quantifiable measurements for TAVI planning.

## 1 Introduction

**Fig. 1:**
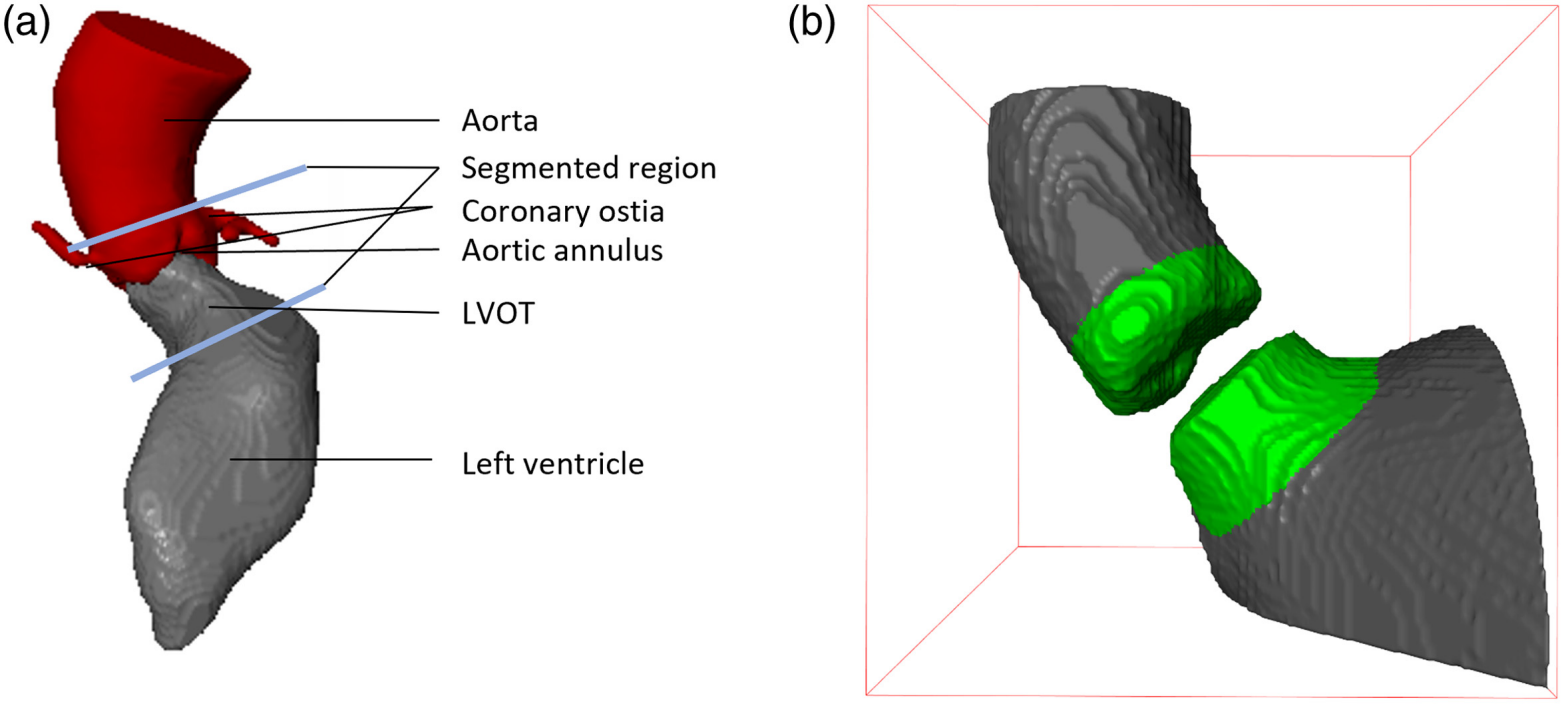
(a) The aortic outflow region is comprised of the composite aortic root and LVOT. (b) The segmentation provided by state-of-the-art solutions, such as TotalSegmentator,^4^ does not completely cover the aortic outflow region (olive).

Transcatheter aortic valve implantation (TAVI) is widely used to treat patients with aortic valve stenosis.^1^ Preoperative image acquisition with computed tomography (CT) is recommended for risk assessment and planning by clinical guidelines.^2^ The aortic root is commonly evaluated using CT image data to derive the annulus diameter and position of the coronary ostia for prosthesis selection. The analysis of the anatomy of the left ventricular outflow tract (LVOT) provides additional relevant information. For example, the angle between the LVOT and ascending aorta was shown to be a risk factor for aortic regurgitation.^3^ Subsequently, we refer to the composite aortic root and LVOT as the aortic outflow region [Fig. 1(a)].

A comprehensive analysis of the aortic root and LVOT requires the extraction of the patient-individual anatomy via segmentation. In previous work, Lalys et al. proposed an aortic root analysis approach starting with detecting the aortic centerline based on a manual marker followed by a segmentation using a deformable three-dimensional (3D) snake.^5^ Conversely, Elattar et al. presented a fully automatic approach based on thresholding and normalized cuts.^6^ However, thresholding is susceptible to image distortions through noise, artifacts, or uneven distribution of contrast agents.^7^

Another line of work uses atlas-based segmentation.^8^^,^^9^ These techniques might have difficulties representing the local variation in pathological aortic root anatomy.^7^

Deep learning (DL) has shown good performance on various segmentation tasks.^10^ Typically, this is formulated as a supervised problem requiring annotated data. Saitta et al.^11^ used semi-automatic region growing for data annotation. For segmentation, they proposed detecting the region of interest (ROI) using template matching followed by segmenting the aortic root using a neural network. However, DL typically requires large amounts of annotated data. Therefore, minimizing the annotation complexity is desirable.

Wasserthal et al.^4^ proposed an iterative annotation approach. They trained a network to propose the initial annotations. The final annotations were used to train the TotalSegmentator that segments multiple anatomic structures on CT images. However, the TotalSegmentator output leaves a gap between the left ventricle and the aorta. Therefore, large parts of the LVOT are missing [Fig. 1(b)].

Generating annotations directly in 3D is a challenging and time-consuming task. Weak annotations^12^ aim at alleviating these issues. Li et al.^13^ proposed sparse annotations only labeling one slice per volume. Cai et al.^14^ proposed annotating multiple perpendicular slices to maximize the data distribution coverage. However, Cai et al.^14^ applied their approach to multiple heart structures, whereas our work solely focuses on the aortic annulus region. We assume that this region has a tubular structure and, therefore, propose annotating two-dimensional (2D) slices perpendicular to the centerline instead. As 2D cross-sectional annotation is well known in clinical practice, our annotation scheme is intended to be intuitive for domain experts.

We present an annotation scheme based on sparse contours. In addition, we propose a deep-learning-based 3D segmentation approach for the aortic outflow region. Using screened Poisson surface reconstruction,^15^ expert-annotated 2D cross-sections are transformed into 3D masks. We use these masks to train a 3D segmentation model. In addition, we show that our fully automatic segmentation approach facilitates reproducible and quantifiable measurements of the aortic outflow region. In contrast to previous works,^5^^,^^6^^,^^8^^,^^9^^,^^11^ we include measurements of the LVOT.

## 2 Methods

**Fig. 2:**
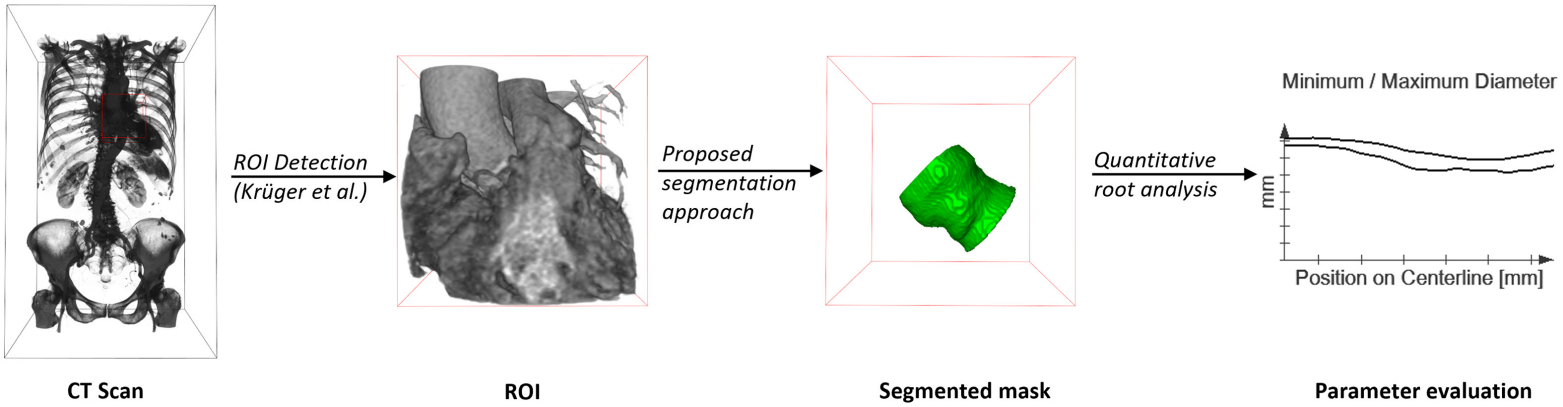
Image analysis pipeline: the CT scan is cropped to the ROI centered around the aortic annulus and coronary ostia.^16^ Our proposed segmentation network takes the cropped image as input and outputs the 3D mask. The segmentation mask is processed to obtain parameters relevant for TAVI planning. CT image data are visualized as volume rendering to provide a better impression of anatomical structures.

The processing workflow of our solution comprises valve region detection, segmentation, and quantitative assessment (Fig. 2). For segmentation, we trained neural networks with representative clinical data sparsely annotated by experts.

### 2.1 Data

**Fig. 3:**
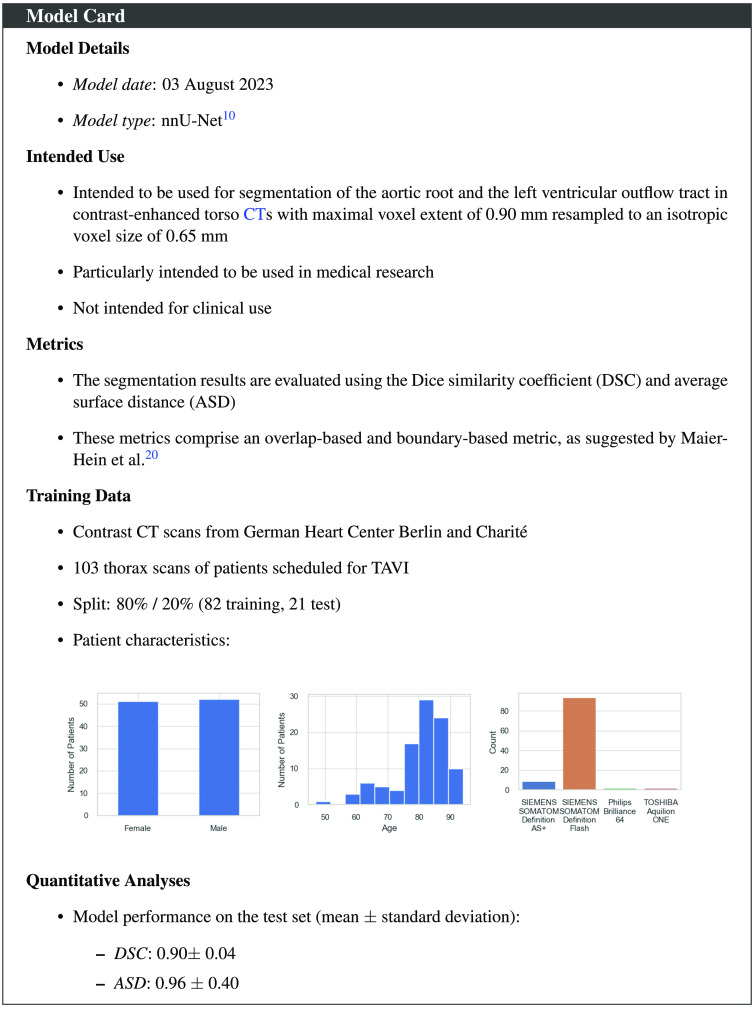
Model card of the proposed segmentation model following the framework proposed by Mitchell et al.^17^

We used a dataset comprising contrast-enhanced CT scans of the torso of 103 patients from the German Heart Center Berlin and the Charité TAVI registry.^16^ Data were acquired by multiple scanner types: SIEMENS SOMATOM Definition Flash, SIEMENS SOMATOM Definition AS+, TOSHIBA Aquilion ONE, and Philips Brilliance 64 (Fig. 3). The acquired images had a resolution between 0.57 and 0.90 mm, with 0.65 mm being the median value. All patients were scanned prior to TAVI. Their average age at acquisition was 80±9 years, and 51 patients were female (Fig. 3). The study was approved by the local ethics committee (EA1/062/19).

#### 2.1.1 Data preparation

**Fig. 4:**
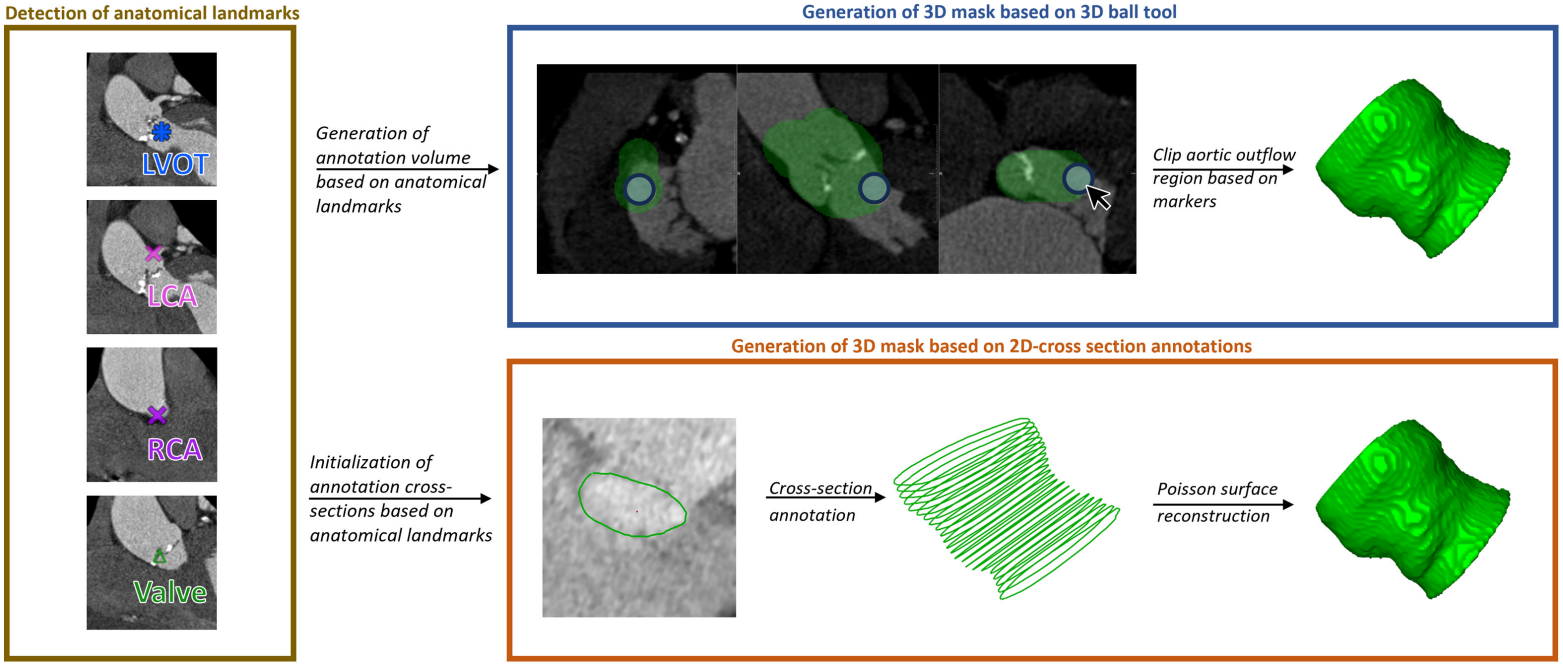
Annotation scheme: landmarks are placed at the LVOT, right coronary artery (RCA), left coronary artery (LCA), and valve. The landmarks indicate the region relevant for aortic outflow region annotation using a 3D ball tool. After 3D annotation, the markers are additionally used to clip the relevant aortic region along the centerline. The proposed 2D cross-section annotation scheme requires the landmarks for centerline definition. Cross-sections are initialized perpendicular to the centerline. After correcting the contours, a 3D mask is generated using screened Poisson surface reconstruction.^15^

We combined the results of a Vesselness filter^18^ and a Hough transform circle detector to calculate a cost image from the CT scan. Based on markers defining LVOT and coronary arteries (Fig. 4) and the cost image, the centerline of the aortic root and LVOT was calculated with the Dijkstra path search. 2D cross-sections were defined perpendicular to the centerline with an offset of 1 mm. As proposed by Kaufhold et al.,^19^ the contours on each cross-section were initialized using a ray-casting approach. On each cross-section, rays pointing outward from the centerline point were defined. Intensity and gradient profiles were extracted along each ray to identify the boundary point. Between the boundary points on each cross-section, the vessel contour was interpolated. Domain experts corrected the contours in multiplanar reconstruction views aligned with the cross-sections’ orientation. Screened Poisson surface reconstruction was applied to create a 3D mask from the 2D cross-sections (Fig. 4).^15^ This algorithm considers the contours to be an oriented point cloud. The point cloud is perceived as the boundary’s normal field. Hence, the surface is retrieved by finding a scalar function with gradients matching the normal field. The isosurface is extracted to find the object’s surface. It is approximated by solving an energy equation that combines a gradient constraint integrated over the spatial domain with a value constraint summed over the given input points. The authors propose the Neumann boundary condition as it has shown good performance in the presence of missing data.^15^ As suggested by Kazhdan et al.,^15^ we set the screening weight to 4, the “bounding-box-scale” to 1.1, and the “samples-per-node” to 1. For surface extraction, the octree depth was set to 8, constituting an adequate resolution and computational complexity.

Eleven datasets were randomly selected and annotated using a mask-based annotation scheme. Domain experts used a 3D ball tool with a modifiable diameter to annotate the aortic outflow region (Fig. 4). We use these annotations to compare the resulting masks with masks obtained from the proposed sparse annotation scheme. The selected 11 datasets were labeled independently by two domain experts using the proposed annotation scheme and the mask-based annotation to facilitate analyses of the inter-observer variance by means of the average surface distance (ASD) and Dice similarity coefficient (DSC).

#### 2.1.2 Segmentation

The masks generated from the sparsely annotated data were used to train segmentation networks. Given a cropped version of the CT images, the networks output 3D masks. Therefore, the images were cropped to the ROI as described by Krüger et al. (Fig. 2).^16^ The ROI is centered around the aortic annulus and coronary ostia with a fixed size of $80\times 80\times 80\;{\mathrm{mm}}^{3}$. However, instead of using the proposed ensemble model, only the model trained with Focal Tversky (0.85) loss was applied to reduce the computational complexity. All cropped images were resampled to an isotropic resolution of 0.65 mm. This corresponds to the median value present in the dataset. We compare the segmentation performance of the nnU-Net^10^ and the Swin UNETR.^20^ The nnU-Net is a framework proposing self-configuring segmentation networks.^10^ We used the default configuration for 3D high-resolution images. The Swin UNETR is a hierarchical vision transformer.^20^ As proposed by the authors, we set the size of the embedding space to 48 and the sliding window overlap to 0.7. The data were split into 80% training and 20% test sets. Hence, 82 image pairs were used for training and validation and 21 for testing. The training was done using fivefold cross-validation.

### 2.2 Aortic Valve Region Assessment

The segmentation masks are used to derive parameters for the quantitative assessment of the aortic valve region. For example, Fig. 2 displays the diameter along the centerline. This information can aid prosthesis selection for TAVI.

We evaluate clinically relevant parameters related to the aortic valve annulus and LVOT, i.e., diameter and area. Therefore, domain experts annotated the valve plane in the CT images. The plane was used to obtain the corresponding mask cross-section. We evaluate the influence of the distance between the 2D cross-sections along the centerline of the proposed annotation technique. In addition, we assess the agreement of expert annotation and model segmentation using Bland–Altman analyses.

## 3 Results

### 3.1 Annotation

We compare the proposed 2D cross-section annotation with annotating 3D masks using a 3D ball tool. Intra- and inter-observer variability is assessed by ASD and DSC on cross-sections of the resulting 3D mask as well as for the 3D mask volumes. Table 1 displays the intra-observer variability between annotations obtained through both approaches for two observers. In addition, the inter-observer variability for the 3D ball tool annotations and 2D cross-section annotation is given.

**Table 1:** Intra-/inter-observer variance: 3D mask annotation using a ball tool (3DB) is compared with the proposed 2D cross-section annotation (2DC). The intra-observer variability between the 3DB and 2DC masks for observer 1 (Obs. 1) and observer 2 (Obs. 2) is displayed. In addition, the inter-observer variability using 3DB and 2DC is shown. For 2DC, the masks are compared in 3D and 2D contours derived from the 3D masks. All values are given as mean ± SD.

	3DB - 2DC		3DB - 3DB	2DC - 2DC (3D)	2DC - 2DC (2D)
	Obs. 1	Obs. 2	Obs. 1 - Obs. 2	Obs. 1 - Obs. 2	Obs. 1 - Obs. 2
DSC ↑	0.82±0.05	0.84±0.03	0.87±0.03	0.94±0.02	0.91±0.04
ASD (mm) ↓	1.95±0.65	1.64±0.50	1.32±0.33	0.68±0.18	1.10±0.39

**Fig. 5:**
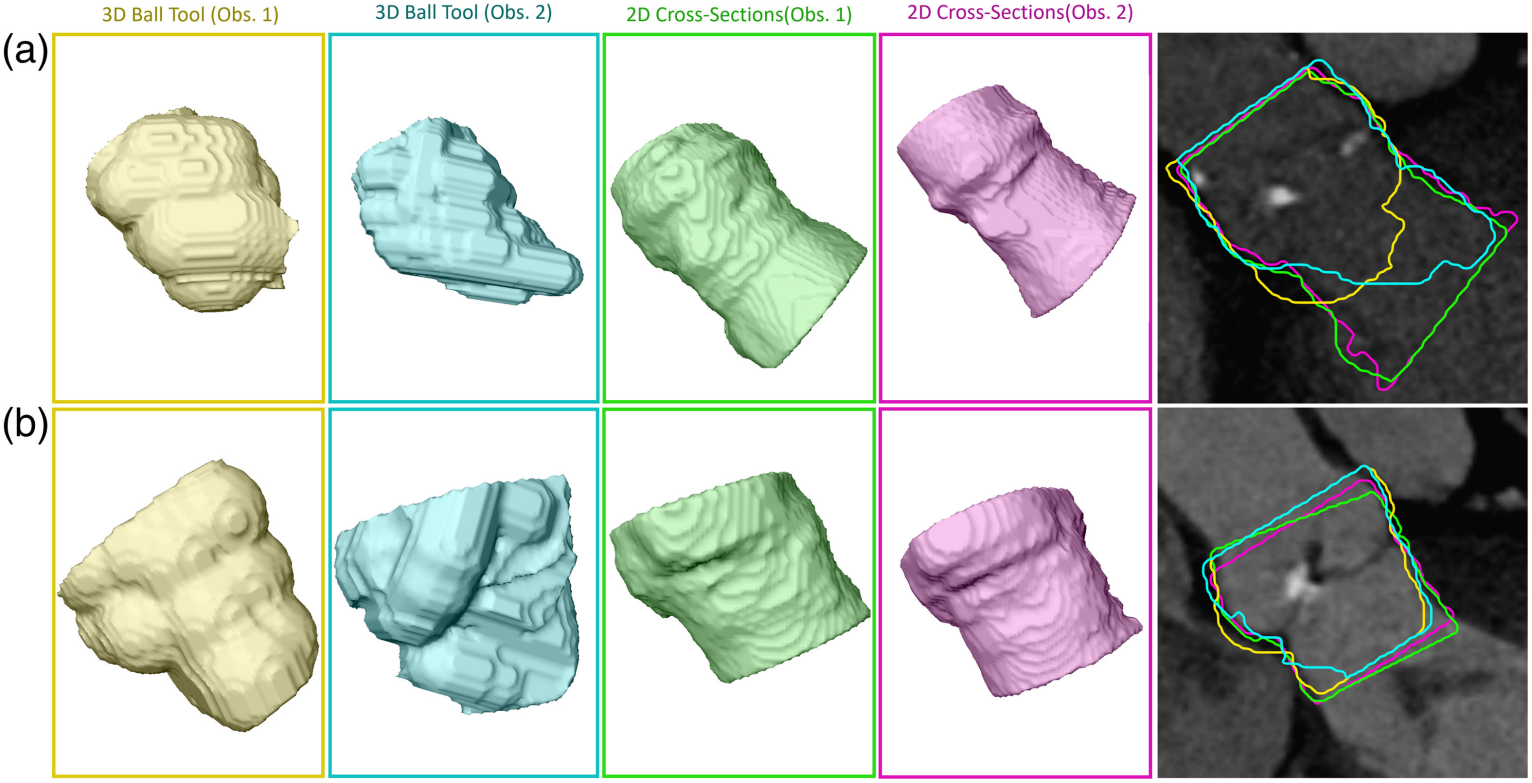
Annotated masks with (a) low pairwise DSC and (b) high DSC. The masks are shown as 3D visualizations. The 2D image viewers present the 2D surface overlay of the masks.

The results show low DSC and high ASD comparing 3D ball tool masks and masks obtained through 2D cross-sections from one observer. The inter-observer variability for 3D ball tool masks results in DSC 0.87±0.03 and ASD 1.32±0.33  mm. Masks obtained from 2D cross-sections result in an improved inter-observer variability. A DSC of 0.94±0.02 and ASD of 0.68±0.18 are achieved. Evaluating the 2D cross-section annotation in 2D, a DSC of 0.91±0.04 and an ASD of 1.10±0.39  mm are obtained. Figure 5 shows an example with high and low intra- and inter-observer variances.

### 3.2 Contour Offset

**Fig. 6:**
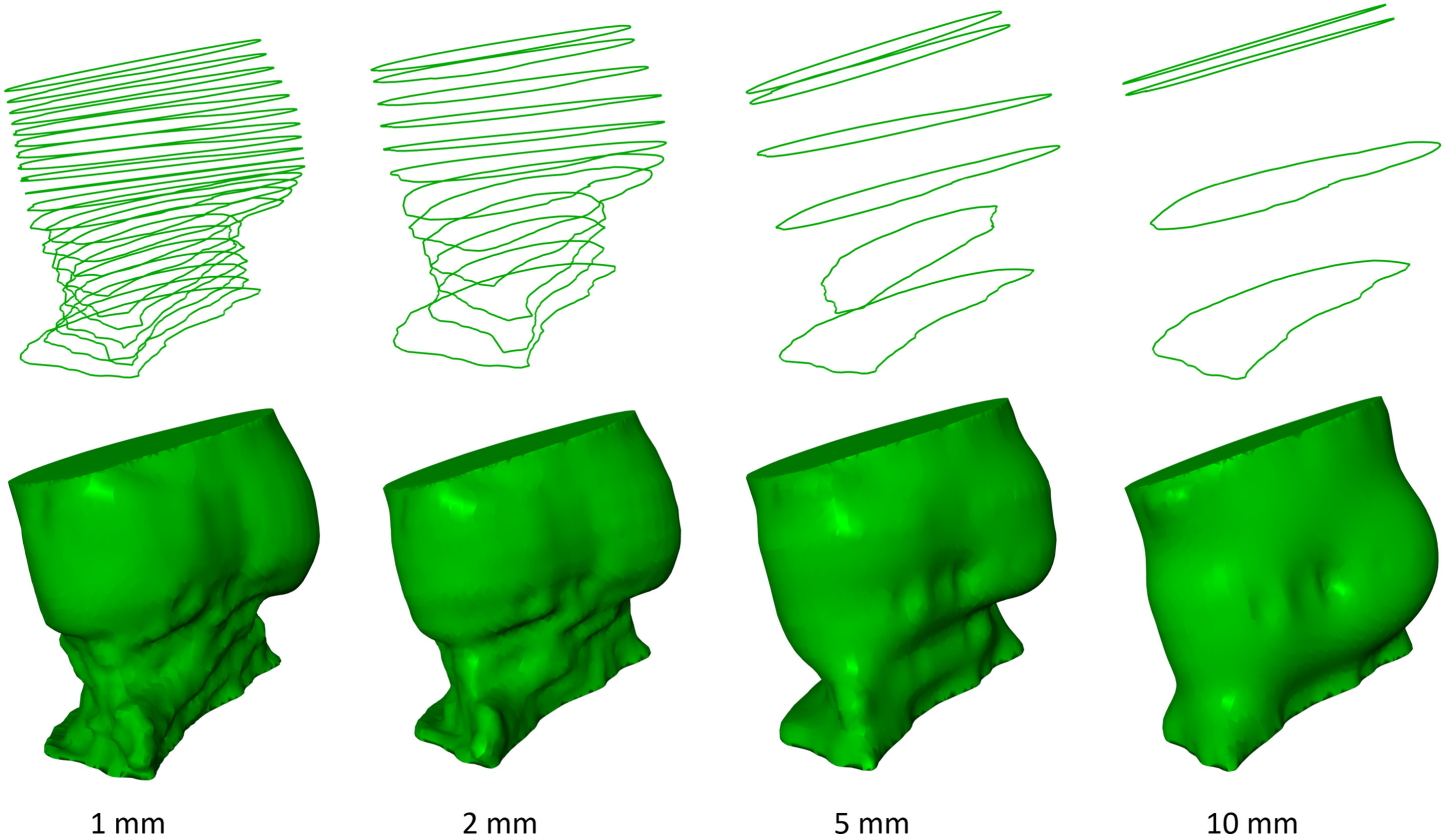
Our proposed annotation scheme comprises cross-sections along the centerline at an offset of 1 mm. By omitting an increasing amount of cross-sections, we obtain cross-sections with offsets of 2, 5, and 10 mm. The first and last contour along the centerline is kept for all masks. The contours (top) and resulting 3D masks (bottom) are shown.

**Fig. 7:**
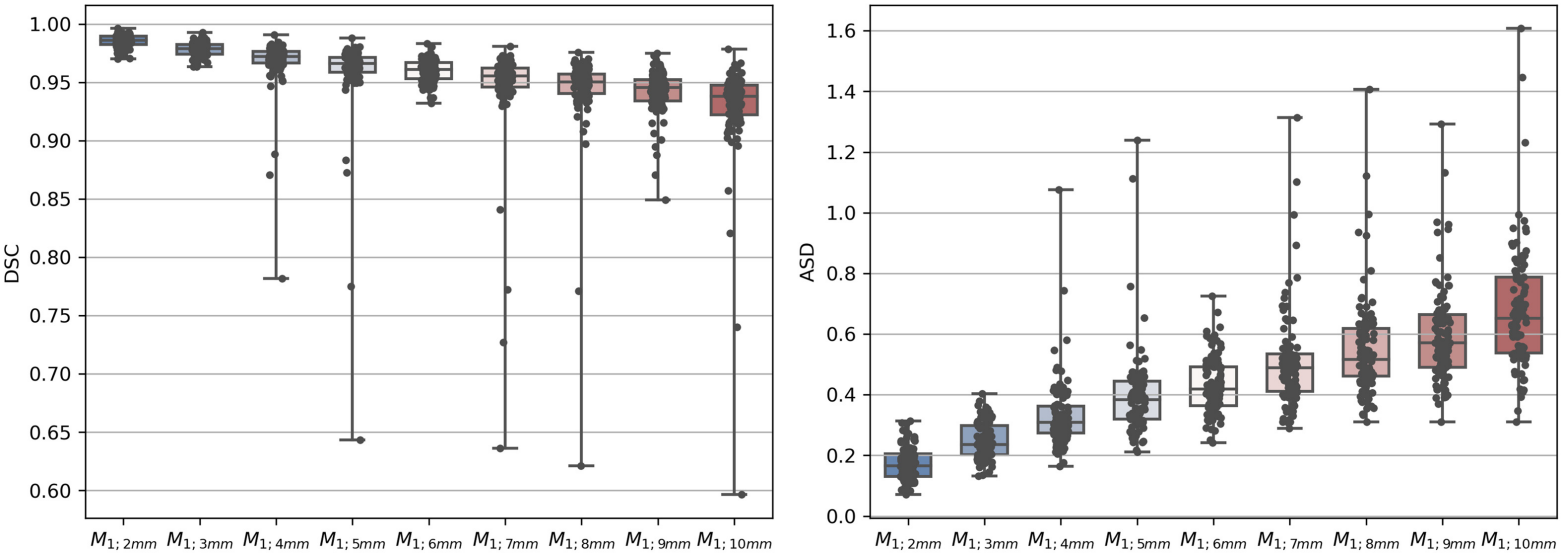
DSC and ASD between $M_{1\;\mathrm{mm}}$ and $M_{2-10\;\mathrm{mm}}$ as boxplot.

The annotated 2D cross-sections were defined with an offset of 1 mm along the centerline. Subsequently, we evaluate the influence of increasing the offset between the cross-sections. Hence, the mask resulting from contours at 1 mm distance ($M_{1\;\mathrm{mm}}$) is compared with the masks resulting from contours at 2 to 10 mm ($M_{2-10\;\mathrm{mm}}$) along the centerline. $M_{1;x\;\mathrm{mm}}$ denotes that masks generated from contours with an offset of 1 and x mm are compared. However, the first and last cross-sections are kept for all masks to ensure that the masks cover the same range along the centerline (Fig. 6). Figure 7 displays the DSC and ASD between $M_{1\;\mathrm{mm}}$ and $M_{2-10\;\mathrm{mm}}$ for the complete dataset.

The DSC and ASD indicate that the similarity between $M_{1\;\mathrm{mm}}$ and $M_{2-10\;\mathrm{mm}}$ decreases with an increasing offset between the contours. The median DSC is close to 1 for $M_{1;2\;\mathrm{mm}}$ and drops below 0.95 for $M_{1;9\;\mathrm{mm}}$ and $M_{1;10\;\mathrm{mm}}$. For $M_{1;5\;\mathrm{mm}}$, the DSC ranges between 0.64 and 0.99. Increasing the offset by 1 mm ($M_{1;6\;\mathrm{mm}}$) reduces the range to 0.93 and 0.98. However, for $M_{1;5\;\mathrm{mm}}$, the majority of data points are between 0.94 and 0.98, and only a few data points result in a low DSC.

**Fig. 8:**
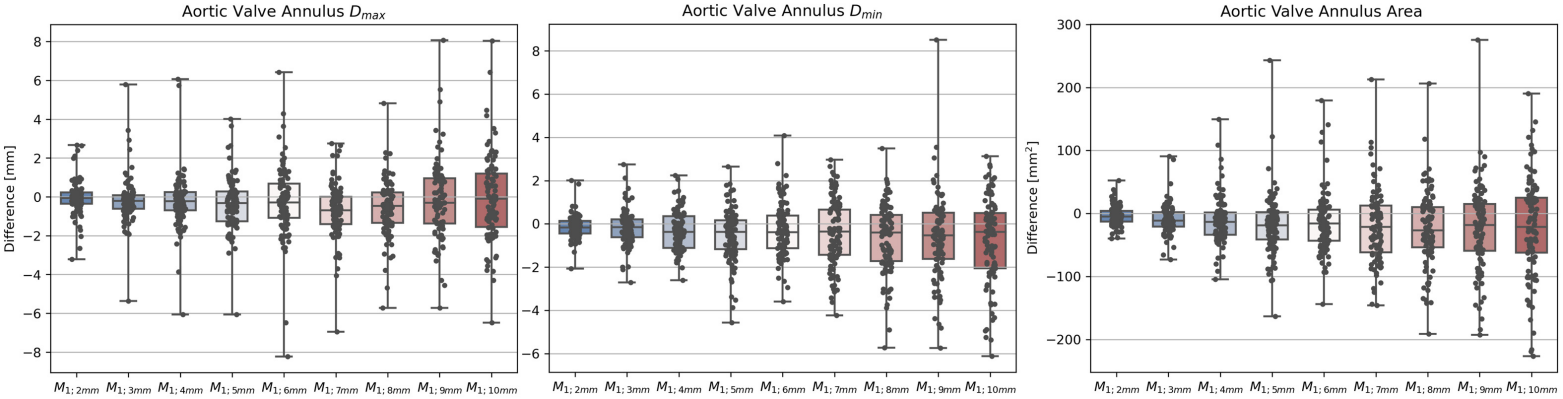
$D_{\max}$, $D_{\min}$, and area difference between $M_{1\;\mathrm{mm}}$ and $M_{2-10\;\mathrm{mm}}$ as boxplot. $M_{1;x\;\mathrm{mm}}$ indicates that masks generated from contours with an offset of 1 and x mm are compared.

Figure 8 shows the influence of increasing the offset between the cross-sections on the aortic valve annulus parameters. It displays the difference between measurements derived from $M_{1\;\mathrm{mm}}$ and $M_{2-10\;\mathrm{mm}}$. The minimum diameter ($D_{\min}$) and area difference gradually decrease for increasing offsets between the contours. This indicates that relevant information about the aortic valve annulus is lost by omitting contours. For the maximum diameter ($D_{\max}$), the median difference decreases between offsets of 2 and 7 mm. Between 7 and 10 mm, the median difference improves slightly. However, the minimum and maximum difference is larger for $M_{1;3-10\;\mathrm{mm}}$ than $M_{1;2\;\mathrm{mm}}$. The variance gradually increases for $D_{\max}$, $D_{\min}$, and the area with an increasing offset between the contours.

### 3.3 Segmentation

The segmentation performance is evaluated using an overlap-based and boundary-based metric, as suggested by Maier-Hein et al.^21^ The DSC^22^ is selected as an overlap-based metric and the ASD^23^ as a boundary-based metric.

Tables 2 and 3 display the segmentation performance for the nnU-Net^10^ and the Swin UNETR^20^ on the validation and testing datasets. Table 2 compares the predicted 3D masks with the 3D masks generated from the 2D annotations (3D-3D). Table 3 analyzes the agreement between the annotated 2D cross-sections and those extracted from the predicted 3D volume (3D-2D). Only cross-sections present in reference and prediction were considered for the 3D-2D ASD.

**Table 2:** 3D masks generated from expert-annotated 2D cross-sections are compared with the predicted 3D masks (3D-3D). The performance on the validation (Val.) and test sets is shown. All values are given as mean ± SD.

	nnU-Net		Swin UNETR	
	Val.	Test	Val.	Test
DSC ↑	0.89±0.05	0.90±0.04	0.85±0.06	0.86±0.06
ASD (mm) ↓	1.16±0.71	0.96±0.40	1.61±0.71	1.61±0.79

**Table 3:** Expert-annotated 2D cross-sections are compared with the same cross-sectional contours resulting from the predicted 3D masks (3D-2D). The performance on the validation (Val.) and test sets are shown. All values are given as mean ± SD.

	nnU-Net		Swin UNETR	
	Val.	Test	Val.	Test
DSC ↑	0.91±0.07	0.92±0.03	0.90±0.08	0.90±0.08
ASD (mm) ↓	1.92±0.93	1.00±0.29	1.16±0.62	1.08±0.52

**Fig. 9:**
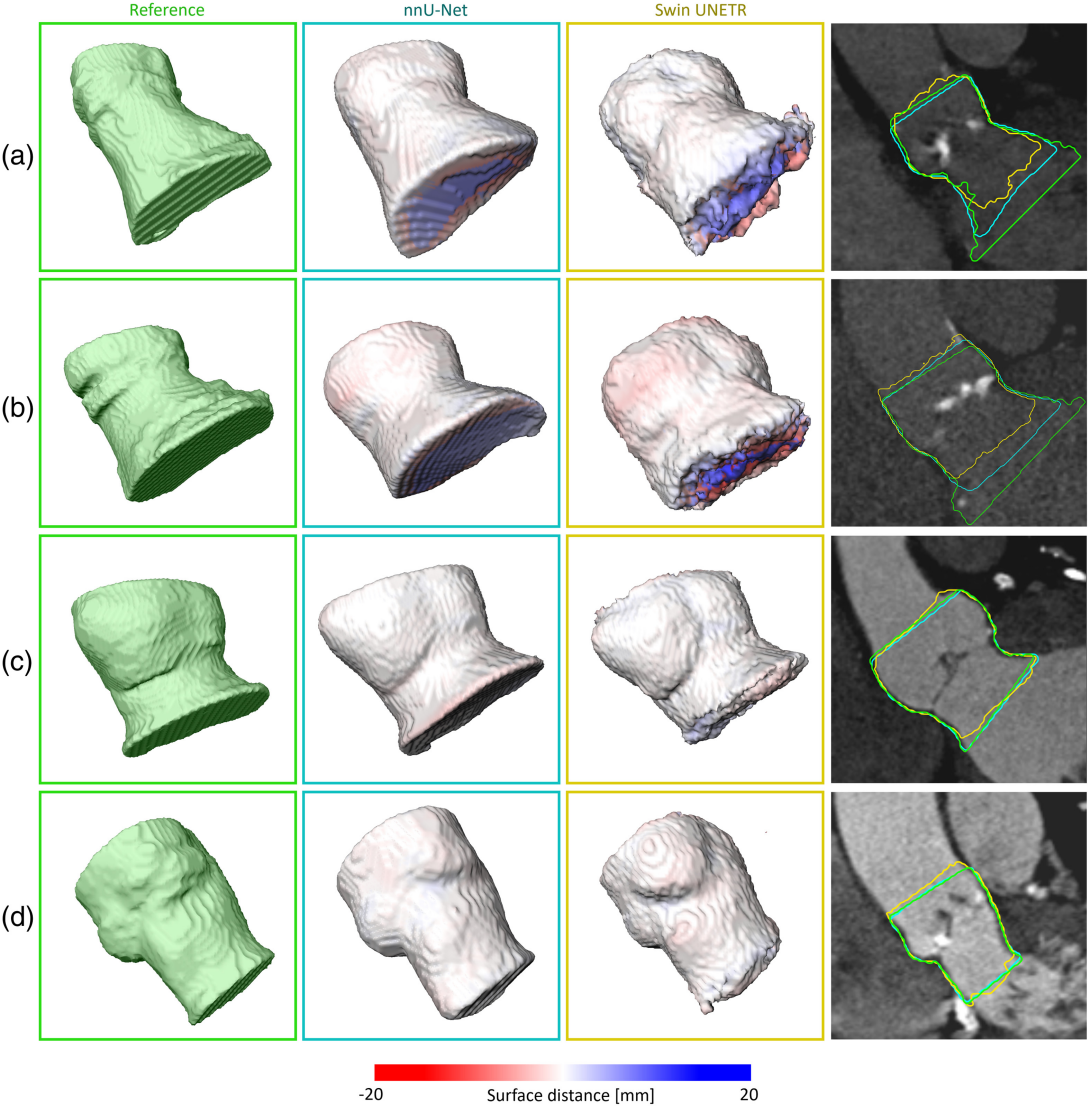
Segmentation results: (a, b) low DSC and (c, d) high DSC between reference masks and model segmentation. 3D visualizations show the reference mask, nnU-Net prediction, and Swin UNETR prediction. The surface distance distribution between reference and prediction is visualized via color coding. The 2D image viewers present the 2D surface overlay of the expert annotated masks generated from 2D cross-sections, nnU-Net prediction, and Swin UNETR prediction.

The nnU-Net achieved a 3D-3D DSC of 0.90 on the test set (Table 2). Hence, the DSC is 0.03 below the inter-observer variance (Table 1). The 3D-3D DSC on the validation data is 0.01 below the DSC on the test set. The 3D-3D ASD on the test set is 0.96 mm. Thus, the ASD mean is 0.28 mm worse than the inter-observer variance and 0.2 mm better than the ASD on the validation set. The Swin UNETR achieves a DSC of 0.85 and 0.86 on the validation and test sets, respectively. Hence, the DSC by the nnU-Net outperformed by 0.04. However, the Swin UNETR ASD is 0.65 mm worse. Figure 9 shows exemplary segmentation results with high and low DSC values.

The nnU-Net 3D-2D DSC is 0.92 on the test set (Table 3) and, hence, is similar to the inter-observer variance (Table 1). The nnU-Net 3D-2D DSC on the validation set is 0.01 below the performance on the test set. The nnU-Net 3D-2D ASD is 1.00 mm on the test set and 1.10 mm for the inter-observer variance. The Swin UNETR performance is similar to the nnU-Net. It achieves a DSC of 0.90 and ASD of 1.08 mm on the test set.

Although the segmentation performance of the nnU-Net and Swin UNETR are similar, the results indicate that the nnU-Net provides better segmentation results. Hence, we consider the nnU-Net for the subsequent evaluations.

### 3.4 Aortic Valve Region Assessment

**Fig. 10:**
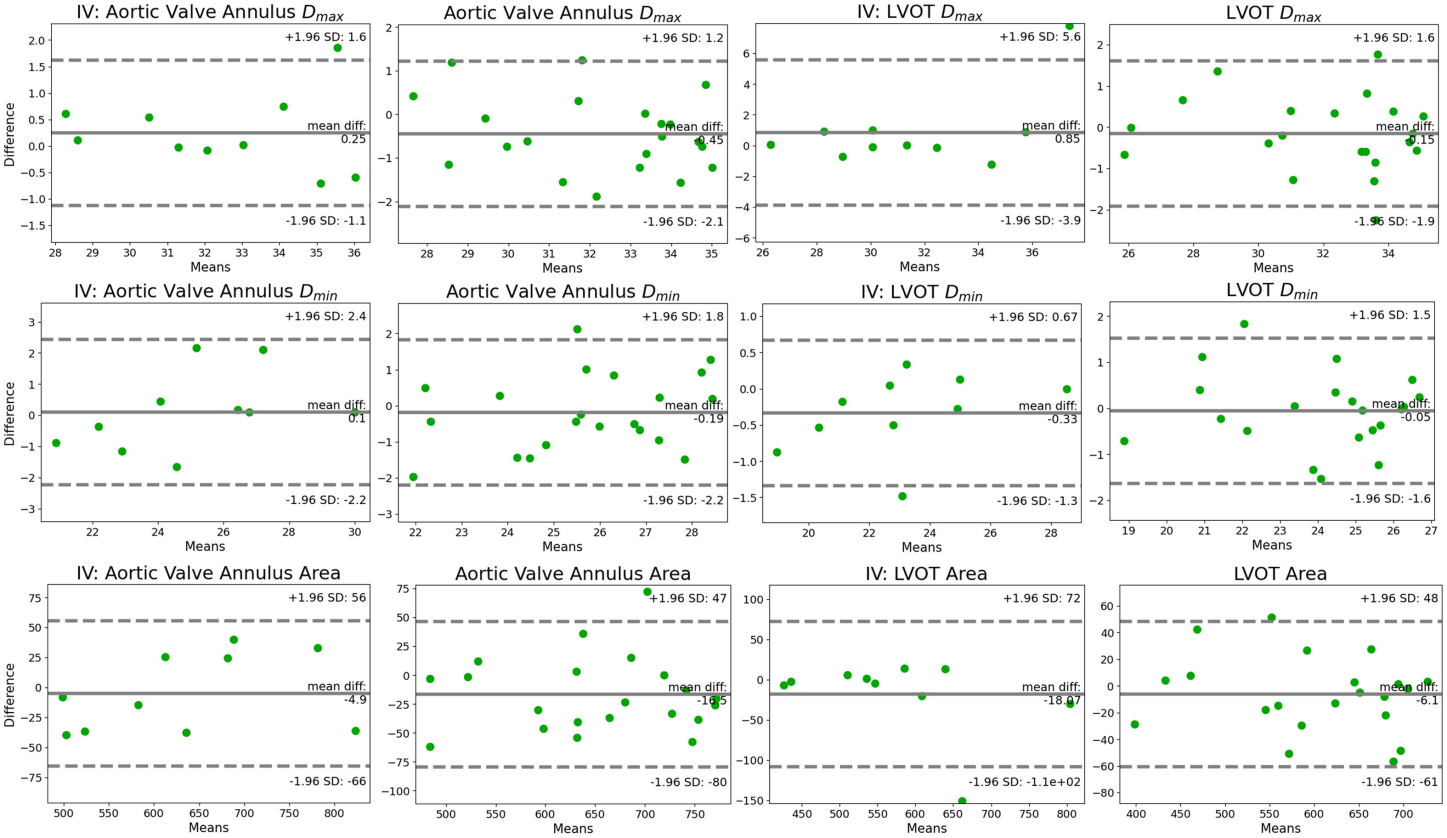
Bland–Altman analysis for the aortic valve annulus and LVOT: the inter-observer variance (IV) and agreement between annotation and prediction are displayed. $D_{\max}$ and $D_{\min}$ are given in mm, and area is in ${\mathrm{mm}}^{2}$.

We evaluate parameters derived from the predicted and annotated aortic outflow region using Bland–Altman analyses. Figure 10 compares $D_{\max}$, $D_{\min}$, and the area measured at the aortic valve annulus and 3 mm below to characterize the LVOT. It analyzes the agreement of the derived parameters from annotation and model prediction. Moreover, it compares the derived parameters from the annotations of two domain experts. We define the diameter as the length of a straight line passing through the center of mass connecting two opposite contour points. Positive difference values hint at larger values measured for the annotation.

For the aortic valve annulus $D_{\max}$, the mean is between 27.5 and 35 mm. The average difference between annotation and prediction is −0.45  mm and between the two observers is 0.25 mm. The limits of agreement for the aortic valve annulus $D_{\max}$ are (−2.1,1.2), (−1.1,1.6) for the inter-observer variance.

**Fig. 11:**
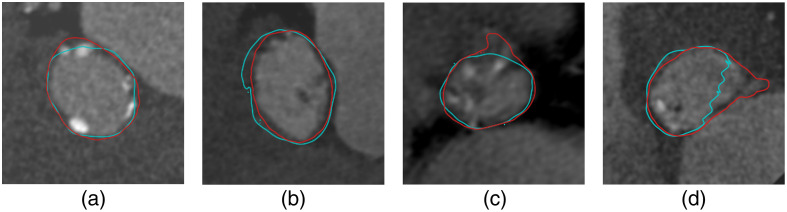
Parameters derived from the depicted contours result in large differences from the reference: (a) LVOT $D_{\max}$ (−2.25  mm), (b) aortic valve annulus $D_{\min}/\text{area}$ ($2.12\;\mathrm{mm}/72.6\;{\mathrm{mm}}^{2}$), (c) aortic valve annulus $D_{\min}$ (−1.96  mm), and (d) LVOT inter-observer-variance area (−151.06  mm). The expert annotation is marked in blue; the predicted contours are in red.

For the aortic valve annulus $D_{\min}$, the mean of measurements ranges between 22 and 28.5 mm. The average $D_{\min}$ difference between annotation and prediction is −0.19  mm and between the two observers is 0.10 mm. The limits of agreement for the aortic valve annulus $D_{\min}$ are (−2.2,1.8), (−2.2,2.4) for the inter-observer variance. Thus, the range between the limits is larger for $D_{\text{min}}$ than for $D_{\max}$. The absolute aortic valve annulus $D_{\min}$ difference is above 1.95 mm in two cases. The corresponding slices are presented in Figs. 11(b) and 11(c). Figure 11(b) suggests that the difference is caused by the annotator not setting the contour at the position with the largest intensity gradient. Figure 11(c) indicates that the segmentation model identified a different contour than the annotator, potentially due to image noise.

At the aortic valve annulus, the area mean of measurements ranges between 460 and $775\;{\mathrm{mm}}^{2}$. The average difference between annotation and prediction is $-16.5\;{\mathrm{mm}}^{2}$ and between the two observers is $-4.9\;{\mathrm{mm}}^{2}$. For the majority of cases, the absolute difference of the measured area is below $35\;{\mathrm{mm}}^{2}$. However, for one case, the difference in measured area is $72.6\;{\mathrm{mm}}^{2}$. This corresponds to one of the cases with a large difference for $D_{\min}$ [Fig. 11(b)].

The LVOT $D_{\max}$ mean of measurements is between 25.8 and 35.1 mm. The average difference between annotation and prediction is −0.15  mm and between the two observers is 0.85 mm. For the majority of cases, the diameter difference is in the range of ±1  mm. However, the largest measured difference for $D_{\max}$ is −2.25  mm. The corresponding slice is shown in Fig. 11(a). The large inter-observer difference is caused by one measurement, resulting in a difference of 7.8 mm. As Fig. 11(d) suggests, the observers identified different LVOT contours. For the LVOT, the $D_{\min}$ means of measurements is between 18.5 and 27 mm. The average difference between annotation and prediction is −0.05 mm and between the two observers is −0.33  mm. The limits of agreement are (−1.6,1.5) for the LVOT $D_{\min}$, (−1.3,0.67) for the inter-observer variance.

The mean of measurements for the LVOT area ranges between 395 and $730\;{\mathrm{mm}}^{2}$. The average difference between annotation and prediction is $-6.1\;{\mathrm{mm}}^{2}$ and between the two observers is $18.07\;{\mathrm{mm}}^{2}$. Despite one outlier [Fig. 11(d)], all absolute inter-observer area differences are below $30\;{\mathrm{mm}}^{2}$.

For all analyses comparing annotation and prediction, the mean is below 0. This suggests that the segmentation model tends to overestimate the volume.

## 4 Discussion

This work combines sparse 2D cross-sectional annotation and point cloud-based surface reconstruction to train a fully automatic 3D segmentation network for the aortic annulus region and the LVOT. In addition, we derive clinically relevant parameters from the segmented masks. We propose a 2D sparse annotation scheme to minimize the annotation complexity for tubular structures. Previous work suggested voxelwise annotation.^11^ By contrast, our approach only requires 2D contours at a given offset along the centerline. The proposed annotation scheme obtains an inter-observer DSC of 0.94 and ASD of 0.68 mm. This indicates that the annotated masks are observer-independent and reproducible. We compared the proposed annotation scheme with 3D mask annotation using a 3D ball tool. The inter-observer variability was inferior to the 2D cross-section annotation. As a 3D ball annotates depth image content that is not visible to the annotator, potentially wrong image content is annotated. By contrast, 2D cross-section annotation allows for displaying all relevant information to the annotator. In addition, 2D cross-sectional views and annotation are well known to domain experts from clinical routines. In Fig. 5(a), the LVOT is annotated as a smaller structure and with higher variance using the 3D ball tool compared with the masks obtained from 2D cross-sections. This indicates that annotating cross-sections along the centerline facilitates reproducible identification of the LVOT boundary. Moreover, the surface of the 3D ball tool masks is bulgy compared with the masks obtained from 2D cross-sections. The results suggest that masks obtained from 2D cross-sections reduce the inter- and intra-observer variances compared with masks generated directly in 3D using a 3D ball tool. In addition, we evaluated the influence of the distance along the centerline between the contours. As expected, the DSC declines with increasing the offset between the annotated 2D cross-sections [Fig. 7 (left)]. This indicates that, by omitting contours, shape information is lost. This results in declining accuracy of the derived parameters (Fig. 8). For example, the aortic valve annulus area difference is $-21.75\pm 63.50\;{\mathrm{mm}}^{2}$ for $M_{1;10\;\mathrm{mm}}$. However, all masks achieve a DSC above 0.9 for $M_{1;6\;\mathrm{mm}}$, whereas four masks are below 0.9 for $M_{1;5\;\mathrm{mm}}$. This hints at the importance of the position of the annotated contours along the centerline. Hence, future research could identify the optimal position of contours to reduce the total number of contours required to obtain accurate aortic outflow region masks. This could additionally reduce the annotation effort. Moreover, annotated 2D cross-sections could be used to train a 2D segmentation network.^24^ Thus, the trained network could predict contours and decrease the offset between contours.

We propose deep-learning-based segmentation. The Swin UNETR reached a DSC of 0.86 and ASD of 1.61 mm on the test set. The nnU-Net outperforms the Swin UNETR and achieves a DSC of 0.90 and ASD of 0.96 mm. Figure 9 suggests that the nnU-Net is superior at detecting the boundaries along the centerline. However, no hyperparameter tuning was performed for the Swin UNETR. Instead, the default parameters suggested by the authors^20^ were used.

Lalys et al.^5^ used deformable 3D snakes for segmentation. They reported an ASD of 1.31 mm. Using an atlas-based segmentation approach, Gao et al.^8^ achieved a DSC of 0.965. By contrast, Saitta et al.^11^ proposed neural network-based segmentation. They reported a DSC of 0.93 and ASD of 1.10 mm. Despite outperforming our approach by 0.03 in terms of DSC, the reported ASD is 0.14 mm worse. The DSC performance difference could be explained by Saitta et al.^11^ considering a larger part of the aorta, thus lowering the proportion of boundary regions. Additionally, Fig. 9(a) indicates that the DSC of our model is diminished by the model detecting different boundaries along the centerline and thus not covering the whole range of annotated cross-sections along the centerline. However, this seems to have a larger effect in the LVOT than in the aorta. This could result from an unclear definition of the lower border of LVOT. As Walmsley stated, there is no line of demarcation indicating the lower border of the tract.^25^ Another reason for diminishing the DSC is that the surfaces of the predicted masks are smoothed compared with the original masks [Fig. 9(a)]. However, the image suggests that the model smooths the random variation introduced by the annotation while keeping the relevant information. Hence, future work could investigate ways to obtain smoother surface annotations from 2D annotations without losing relevant information.

Deriving clinically relevant parameters from segmented 3D masks has been proposed in multiple previous works.^5^^,^^6^^,^^9^^,^^11^ Gao et al.^9^ reported a $D_{\max}$ difference at the aortic valve annulus of 0.7±2.3  mm for observer 1 and 0.6±2.8  mm for observer 2 using an atlas-based segmentation approach. This was outperformed by the neural network-based approach introduced by Saitta et al.^11^ They achieved aortic valve annulus $D_{\max}$ differences of 0.51±1.69  mm. Elattar et al.^6^ suggested thresholding and normalized cuts for segmentation. However, they evaluated the aortic annulus radius instead of $D_{\max}$. For observer 1, a radius difference of 0.24±0.70  mm was reported; for observer 2, it was 0.37±0.82  mm. The diameter being double the radius indicates that our approach outperforms the previous approaches. However, our approach does not involve detecting the aortic valve annulus automatically.

## 5 Conclusion

This work combined sparse 2D cross-sectional annotation and point cloud-based surface reconstruction to train a fully automatic 3D segmentation network for the aortic annulus region and the LVOT. The proposed 2D cross-sectional annotation results in high inter-observer agreement (DSC 0.94). Our segmentation model achieved a DSC of 0.90 and an ASD of 0.96 mm for annotations based on cross-sections sampled with a 1 mm distance along the centerline. Our results suggest that the model segmentations facilitate accurate and reliable measurements of clinically relevant parameters. In future work, we want to evaluate the influence of the LVOT anatomy on the outcome of TAVI.

## Data Availability

The code is not publicly available.

## References

[1] Carroll J. D. et al., “STS-ACC TVT registry of transcatheter aortic valve replacement,” J. Am. Coll. Cardiol. 76, 2492–2516 (2020). JACCDI 0735-1097 10.1016/j.jacc.2020.09.595 33213729

[2] Otto C. M. et al., “2020 ACC/AHA guideline for the management of patients with valvular heart disease: a report of the American College of Cardiology/American Heart Association Joint Committee on Clinical Practice Guidelines,” Circulation 143, E72–E227 (2021). CIRCAZ 0009-7322 10.1161/CIR.0000000000000932 33332150

[3] Sherif M. A. et al., “Anatomic and procedural predictors of paravalvular aortic regurgitation after implantation of the medtronic corevalve bioprosthesis,” J. Am. Coll. Cardiol. 56, 1623–1629 (2010). JACCDI 0735-1097 10.1016/j.jacc.2010.06.035 21050971

[4] Wasserthal J. et al., “TotalSegmentator: robust segmentation of 104 anatomic structures in CT images,” Radiol.: Artif. Intell. 5(5), e230024 (2023). 37795137 10.1148/ryai.230024 PMC10546353

[5] Lalys F. et al., “Automatic aortic root segmentation and anatomical landmarks detection for TAVI procedure planning,” Minimally Invas. Ther. Allied Technol. 28, 157–164 (2019). 10.1080/13645706.2018.1488734 30039720

[6] Elattar M. et al., “Automatic aortic root landmark detection in CTA images for preprocedural planning of transcatheter aortic valve implantation,” Int. J. Cardiovasc. Imaging 32, 501–511 (2016). 10.1007/s10554-015-0793-9 26498339 PMC4751164

[7] Sharma N. et al., “Automated medical image segmentation techniques,” J. Med. Phys. 35, 3 (2010). 10.4103/0971-6203.58777 20177565 PMC2825001

[8] Gao X. et al., “Automatic aortic root segmentation in CTA whole-body dataset,” Proc. SPIE 9785, 97850F (2016). PSISDG 0277-786X 10.1117/12.2216734

[9] Gao X. et al., “Quantification of aortic annulus in computed tomography angiography: validation of a fully automatic methodology,” Eur. J. Radiol. 93, 1–8 (2017). EJRADR 0720-048X 10.1016/j.ejrad.2017.04.020 28668401

[10] Isensee F. et al., “nnU-Net: a self-configuring method for deep learning-based biomedical image segmentation,” Nat. Methods 18, 203–211 (2021).1548-7091 10.1038/s41592-020-01008-z 33288961

[11] Saitta S. et al., “A CT-based deep learning system for automatic assessment of aortic root morphology for TAVI planning,” Comput. Biol. Med. 163, 107147 (2023). CBMDAW 0010-4825 10.1016/j.compbiomed.2023.107147 37329622

[12] Tajbakhsh N. et al., “Embracing imperfect datasets: a review of deep learning solutions for medical image segmentation,” Med. Image Anal. 63, 101693 (2020). 10.1016/j.media.2020.101693 32289663

[13] Li S. et al., “PLN: parasitic-like network for barely supervised medical image segmentation,” IEEE Trans. Med. Imaging 42, 582–593 (2023). ITMID4 0278-0062 10.1109/TMI.2022.3211188 36178993

[14] Cai H. et al., “3D medical image segmentation with sparse annotation via cross-teaching between 3D and 2D networks,” Lect. Notes Comput. Sci. 14222, 614–624 (2023). LNCSD9 0302-9743 10.1007/978-3-031-43898-1_59

[15] Kazhdan M. Hoppe H., “Screened poisson surface reconstruction,” ACM Trans. Graphics 32, 1–13 (2013). ATGRDF 0730-0301 10.1145/2487228.2487237

[16] Krüger N. et al., “Cascaded neural network-based CT image processing for aortic root analysis,” Int. J. Comput. Assist. Radiol. Surg. 17, 507–519 (2022). 10.1007/s11548-021-02554-3 35066774 PMC8873075

[17] Mitchell M. et al., “Model cards for model reporting,” in Proc. Conf. Fairness, Acc., and Transp., ACM, New York, pp. 220–229 (2019).

[18] Frangi A. F. et al., Multiscale Vessel Enhancement Filtering, Vol. 1496, pp. 130–137, Springer Verlag (1998).

[19] Kaufhold L. et al., “Image-based assessment of uncertainty in quantification of carotid lumen,” J. Med. Imaging 5, 034003 (2018). JMEIET 0920-5497 10.1117/1.JMI.5.3.034003 PMC6152582 30840745

[20] Hatamizadeh A. et al., “Swin UNETR: Swin transformers for semantic segmentation of brain tumors in MRI images,” Lect. Notes Comput. Sci. 12962, 272–284 (2022). LNCSD9 0302-9743 10.1007/978-3-031-08999-2_22

[21] Maier-Hein L. et al., “Metrics reloaded: recommendations for image analysis validation,” Nat. Methods 21, 195–212 (2022). 10.1038/s41592-023-02151-z PMC11182665 38347141

[22] Dice L. R., “Measures of the amount of ecologic association between species,” Ecology 26, 297–302 (1945). ECGYAQ 0094-6621 10.2307/1932409

[23] Beneš M. Zitová B., “Performance evaluation of image segmentation algorithms on microscopic image data,” J. Microsc. 257, 65–85 (2015). JMICAR 0022-2720 10.1111/jmi.12186 25233873

[24] Rahlfs H. et al., “Learning carotid vessel wall segmentation in blackblood MRI using sparsely sampled cross-sections from 3D data,” Proc. SPIE 12971, 129271P (2024). PSISDG 0277-786X 10.1117/12.3008294 PMC11245174 39006308

[25] Walmsley R., “Anatomy of left ventricular outflow tract,” Heart 41, 263–267 (1979). 10.1136/hrt.41.3.263 PMC482024 426974

